# The Identification of Immunological Biomarkers in Kidney Cancers

**DOI:** 10.3389/fonc.2018.00456

**Published:** 2018-11-02

**Authors:** Antonio Lopez-Beltran, Vanessa Henriques, Alessia Cimadamore, Matteo Santoni, Liang Cheng, Thomas Gevaert, Ana Blanca, Francesco Massari, Marina Scarpelli, Rodolfo Montironi

**Affiliations:** ^1^Department of Pathology and Surgery, Faculty of Medicine, Cordoba University, Cordoba, Spain; ^2^Pathology Service, Champalimaud Clinical Center, Lisbon, Portugal; ^3^Section of Pathological Anatomy, United Hospital, School of Medicine, Polytechnic University of the Marche Region, Ancona, Italy; ^4^Oncology Unit, Macerata Hospital, Macerata, Italy; ^5^Department of Pathology and Laboratory Medicine, Indiana University School of Medicine, Indianapolis, IN, United States; ^6^Laboratory of Experimental Urology, Organ Systems, KU Leuven, Leuven, Belgium; ^7^Department of Pathology, AZ Klina, Brasschaat, Belgium; ^8^Instituto Maimonides de Investigación Biomédica de Córdoba, Córdoba, Spain; ^9^Division of Oncology, S. Orsola-Malpighi Hospital, Bologna, Italy

**Keywords:** renal cell carcinoma, PD-L1, immunotherapy, RCC subtypes, immunological biomarker, predictive biomarker, tumor mutation load

## Abstract

The recent approval of several agents have revolutionized the scenario of therapeutic management of metastatic renal cell carcinoma (RCC) allowing us to reach important clinical end points with extended patients' survival. Actually, every new drug approved has represented an important step forward to the improvement of patient's survival. On the other hand, we now understand that RCC includes a large group of tumor entities, each of them with different genetic and mutational alterations, but also showing different clinical behavior; a reason behind the needs of subtype specific personalized approach to therapy of RCC. Immunotherapy is gradually becoming a key factor in the therapeutic algorithm for patients with locally advanced or metastatic RCC. Due to the combination of potent treatment success and potentially deadly adverse effects from immune checkpoint inhibitors (ICI), gathering prognostic and predictive information about FDA-indicated tumors seems to be prudent. Robust and reliable biomarkers are crucial for patient's selection of treatments with immunomodulatory drugs. PD-L1 expression is a poor prognostic factor and predictive of better responses from both PD-1 and PD-L1 inhibitors in a variety of tumor types including RCC. Each FDA approved PD-1/PD-L1 drug is paired with a PD-L1 Immunohistochemistry (IHC) assay. Thus, there is need for improved knowledge and application of PD-1/PD-L1 IHC biomarkers in daily practice. IHC staining appears in membranous fashion. The atezolizumab approved IHC assay is unique in that only immune cell staining is quantified for the use of this assay in RCC. A single biomarker for patient selection may not be feasible, given that immune responses are dynamic and evolve over time. Biomarker development for ICI drugs will likely require integration of multiple biologic components like PD-L1 expression, TILs and mutational load. New methodological approaches based on digital pathology may be relevant since they will allow recognition of the biomarker and to objectively quantitate its expression, and therefore might produce objective and reproducible cut-off assessment. Multidisciplinary approach is very much needed to fully develop the current and future value of ICI in clinical practice.

## Introduction

The recent approval of several agents have revolutionized the scenario of therapeutic management of metastatic renal cell carcinoma (RCC) allowing us to reach important clinical end points with extended patients' survival (1).

The first generation of immune checkpoint inhibitors (anti-CTLA-4 and anti-PD-1/PD-L1) targeted natural immune homeostasis pathways to drive anti-tumor immune responses. These agents led to unprecedented results in patients with previously incurable metastatic disease and therefore became first-line therapies for some advanced cancers (2–12). Since these agents are efficacious in only a minority of patients, however, newer strategies are becoming available that target additional immunomodulatory mechanisms to activate patients' own anti-tumor immune responses. Emerging targets include co-inhibitory and co-stimulatory markers of the innate and adaptive immune system.

In this review, we will discuss: (1) Pathologic and molecular subtypes of RCC; (2) Current landscape of targeted therapy in renal cell carcinoma; (3) Overview of immunotherapy in renal cell carcinoma; (4) Predictive immunological biomarkers in renal cell carcinoma; (5) Gene expression as predictive biomarkers in renal cell carcinoma; (6) The current status of PD-L1 immunohistochemistry; (7) MMR-deficiency and mutational load in RCC; and (8) Biomarkers of acquired resistance. Finally, we briefly highlight likely future perspectives of predictive biomarkers of immunotherapy in RCC.

## Pathologic and molecular subtypes of renal cell carcinoma

Clear cell RCC (ccRCC) accounts for about 75% of kidney cancer while the other 25% are classified as non-clear cell renal cell carcinoma (nccRCC) (13). Over a dozen pathological subtypes are now recognized by the most recent World Health Organization classification of Tumors of the Urinary System and Male Genital Organs (13). These subtypes include papillary renal cell carcinoma (pRCC) (20%) and chromophobe renal cell carcinoma (chRCC) (5%), which are the most frequent nccRCC subtypes; hereditary leiomyomatosis and renal cell associated -carcinoma, collecting duct carcinoma, renal medullary carcinoma, MiT family translocation carcinoma, succinate dehydrogenase-deficient RCC, mucinous tubular and spindle cell carcinoma, tubulocystic RCC, Acquired cystic disease-associated RCC, clear cell papillary RCC, and RCC unclassified represent less common subtypes (13).

Several genomic changes have been found in ccRCC, mostly epigenetic reprogramming and oncogenic metabolism pathways alterations (13–18) with other common genetic changes in genes controlling cellular oxygen pathway (e.g., VHL) and the maintenance of chromatin structure (e.g., PBRM1) (19–22). TCGA analysis of a ccRCC cohort found similar genomic changes and reported recurrent alterations in the PI(3)K/AKT pathway and several epigenetic changes in DNA methylation (22). Molecular stratification of ccRCC revealed 2 different subtypes: clear cell type A (ccA) and B (ccB), with ccA patients having a markedly better prognosis (23, 24). A second TCGA study focussed on papillary RCC (pRCC) and found that type 1 and type 2 pRCC are distinctly different diseases based on molecular features and that type 2 pRCC is a heterogeneous disease with at least three different subgroups (25). A third TCGA project focussed on the chromophobe RCC (ChRCC) and found gene expression changes related to mitochondrial function and recurrent structural breakpoints within TERT promoter region (26). Recently, a multilevel molecular characterization of the 3 TCGA RCC databases revealed nine major genomic RCC subtypes, each one being distinct in terms of altered pathways and patient survival (16). Overlapping and subtype-specific genomic changes were observed, and good correlation with histologic subtypes was noticed. These molecular classes show substantial molecular diversity represented within each major histologic type, but importantly, actionable alterations also included PI3K and immune checkpoint pathways (16).

## Current landscape of targeted therapy in renal cell carcinoma

The better knowledge of molecularly altered pathways of RCC has led to the development of new classes of drugs rising the targeting therapy era (1, 12, 16–23, 27). Angiogenesis, the hallmark of RCC, is the final target of several TKi (Sunitinib, Axitinib, Sorafenib and pazopanib) (1–12). After angiogenesis, the finding that, the deregulation of the PI3K–Akt–mTOR pathway, activated at different levels of the signaling cascade, drives RCC progression has led to the development of the mTOR inhibitors everolimus and temsirolimus. The association between everolimus and lenvatinib (a VEGFR1, VEGFR2 and VEGFR3 FGFR1, FGFR2, FGFR3, FGFR4, PDGFR, RET and KIT inhibitor) has been recently explored in a phase II clinical trial which demonstrated a better progression free survival (PFS) for patients receiving the combination of these two drugs compared to those who received everolimus monotherapy (10). Recently, also the mesenchymal-epithelial transition and multi-tyrosine kinases inhibitor cabozantinib has been included in clinical practice (1–12).

These drugs have led to an improvement in overall survival (OS) (sunitinib, pazopanib, cabozantinib, temsirolimus) and PFS (sunitinib, axitinib, cabozantinib, sorafenib, pazopanib, everolimus and temsirolimus) showing a safety profile with a remarkable clinical activity in a disease which has always been poor of active treatments (12, 15).

## Overview of immunotherapy in renal cell carcinoma

Targeting drugs have significantly changed the course of RCC, but it's likely that a new classes of agents, the immune-checkpoint inhibitors (ICI), are destined to feed this new paradigm in RCC treatment (12, 28–51).

**Figure 1:**
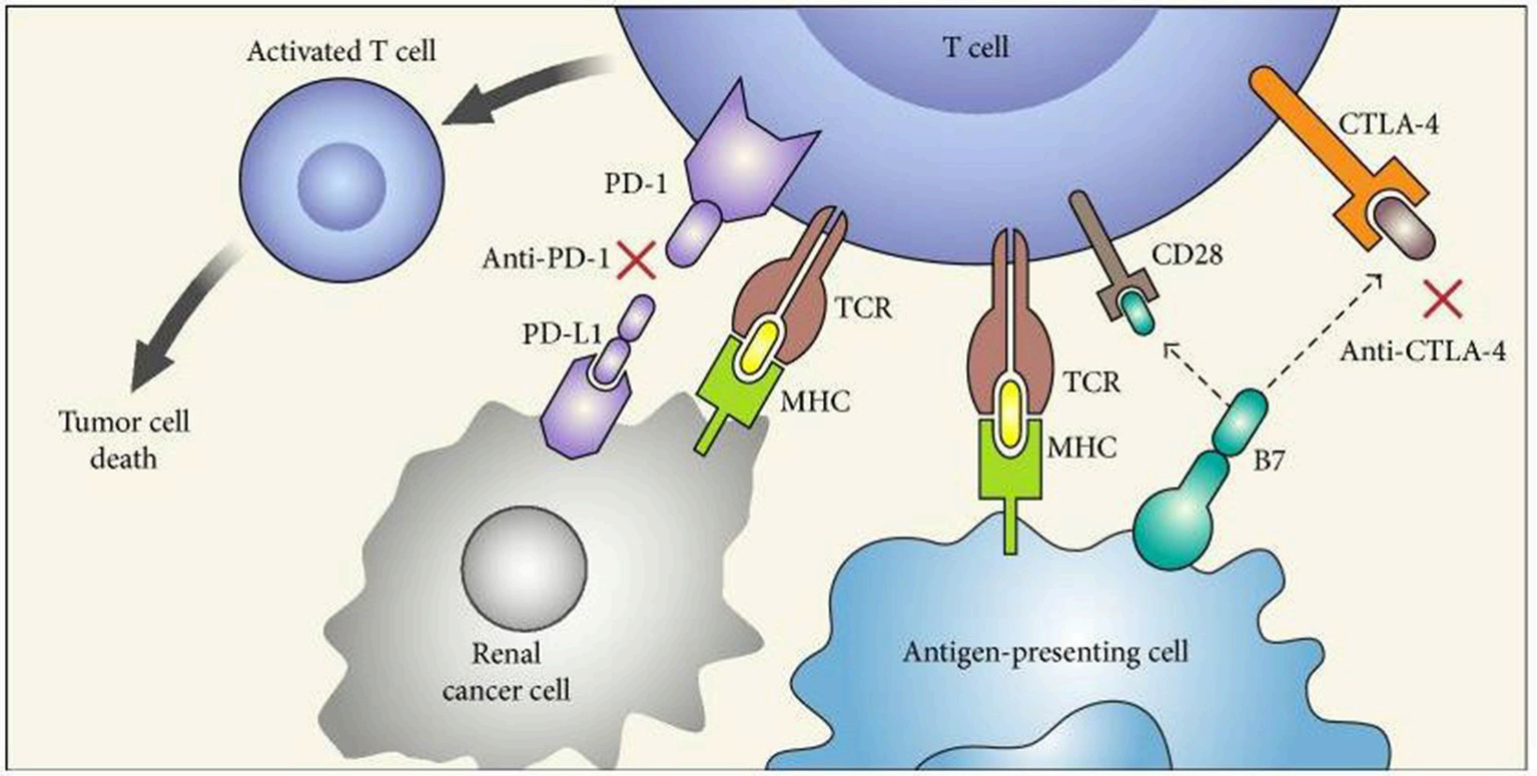
“Mechanism of action of immune checkpoint inhibitors. PD-1 is expressed on activated T cells and when it binds to its ligand PD-L1 on tumor cells leads to T cell exhaustion. CTLA-4 competes with CD28 (costimulatory T cell molecule) for B7 ligands (CD80 and CD86 that are not shown in the figure) and upon activation decreases T cell proliferation as well as activity. Blockade of CTLA-4 (by anti-CTLA-4) and PD-1 (anti-PD-1) or PD-L1 stimulates effector T cells to produce antitumor responses. PD-1, programmed death-1; PD-L1, programmed death-ligand 1; MHC, major histocompatibility complex; TCR, T cell receptor; and CTLA-4, cytotoxic T lymphocyte antigen.” Reproduced from Raman and Vaena (52). Available via license: CC BY 3.0.

Programmed Death Receptor 1/Programmed Death Receptor Ligand 1 (PD 1/PD-L1) and Cytotoxic T Lymphocytes Antigen 4 (CTLA-4) inhibitors are agents able to target specific pathways related to immune-response which are often hyper-activated by tumor cell interaction (46). By inhibition of these targets, ICI could reactivate a specific immune response against tumor cells (Figure 1) (52). The observation that, RCC is related to a high mutation burden and so maybe to a high antigens expression, has led to test these drugs in different stages of the disease. Checkmate 025 was the first large phase III clinical trials comparing the PD-1 inhibitor nivolumab to everolimus in patients with locally advanced or metastatic RCC progressed to at least one VEGF/VEGFR inhibitor (11). This study met its primary endpoints showing an OS benefit in patients receiving nivolumab. Furthermore, patients treated with immunotherapy showed a higher overall response rate (ORR) compared to everolimus with an important percentage of patients achieving long lasting response (11). It is not surprising that the important results achieved in this trial have move to explore immunotherapy in other setting, such as adjuvant/neo-adjuvant stage and as first line therapy (12). Two different strategies been adopted: (1) the combination between an immune-checkpoint inhibitor and a VEGF inhibitor has been evaluated in a phase II trials. Indeed, in Immotion150 305 patients with locally advanced/mRCC and untreated RCC were randomized to receive: atezolizumab (an anti PD-L1 inhibitor) and bevacizumab, atezolizumab alone or sunitinib (41). The association arm resulted in a longer PFS compared to atezolizumab (6.1 months) and sunitinib arms with a higher percentage of ORR in combination arm (41). Of note, patients with PD-L1 positive expression (≥1%) showed a longer PFS (14.7 months) and higher ORR (46%) in atezolizumab arm; (14) and (2) the combination between two immune-checkpoint inhibitors have been recently tested in a large phase III trial: The Checkmate 214. In this study patients were randomized to receive the nivolumab (anti PD-1) and Ipilimumab (Anti CTLA 4) combination or sunitinib as first line therapy (42). In ESMO 2017, Escudier et al. (42) presented primary results after 17.5 months of follow up showing that the combination between ipilimumab-nivolumab resulted in higher ORR and complete response rate in intermediate/poor risk patients. Of note, patients with intermediate/poor risk disease and PD-L1 expression ≥1% showed higher ORR and PFS compared to sunitinib, while patients with favorable category of risk (showing lower PD-L1 expression) displayed a longer PFS and a higher ORR with sunitinib (42) (Table 1).

**Table 1:** Results obtained in selected trials exploring immune check point inhibitors in metastatic/locally advanced RCC using different combination of drugs.

**Study name with experimental and comparator arms**	**Setting**	**NITT**	**NPD-L1+**	**OSITT**	**HR**	**OSPD-L1+**	**PFSITT**	**HR**	**PFSPD-L1+**	**HR**	**ORRITT**	**ORRPD-L1+**	**CR**
**IMMOTION150**													
Atezolizumab + Bevacizumab	Untreated patients with locally advanced or metastatic renal cell carcinoma	101	164	NR	NR	NR	NR	NR	NR	NR	32%	46%	NR
Atezolizumab		103		NR		NR	NR		NR		25%	28%	NR
Sunitinib		101		NR		NR	NR		NR		29%	27%	NR
**CHECKMATE 214**													
Ipilimumab + Nivolumab	Untreated patients with locally advanced or metastatic renal cell carcinoma	550	204	NR	NR	NR	11.6	0.82	22.8^*^	0.48	NR	58%^*^	9.4%^*^
Sunitinib		546	224	NR	NR	NR	8.4		5.9^*^		NR	25%^*^	1.2%^*^

NR, not reported; PFS: progression free survival; OS, overall survival; ORR, overall response rate; CR, complete response.

* *Intermediate/poor risk patients with PD-L1 expression ≥1%*.

These encouraging results suggest that we are about to enter a new era for the management of metastatic RCC since the data provided from these trials might represent only the tip of the iceberg, and therefore, we could expect more therapeutic novelties to come. Nonetheless, even if immunotherapy provides a new hope for patients with metastatic RCC, the “old” targeting therapy is far from being abandoned. Indeed, Checkmate 025 showed that nivolumab is better than everolimus, but there are other agents showing to be extremely effective after VEGF/VEGFR inhibitors progression, thus, the decision of second line treatment should be weighted on the basis of the clinical outcome pursued as well as patient preference and toxicity profile (53). Though in first line setting immunotherapy showed interesting results, it's probably that the positive effect could be restricted to patients with specific clinical features, such as intermediate/poor risk disease while patients with favorable profile could benefit from a standard therapeutic approach (46). It is probably that the worst clinical profile of the disease could be related to a high mutational burden of tumor cells, therefore resulting in a higher antigens expression. Preliminary data seems to indicate that these patients with high mutational load present a higher percentage of tumors with positive PD-L1 expression. Future studies will help us to better understand the role of PD-L1 as prognostic and predictive response factor since to date we have highly diverging information. Indeed, a meta-analysis of six published studies revealed that a higher level of PD-L1 expression increased the risk of death by representing therefore a negative prognostic factor (44). Differently to what was expected, the improved OS with nivolumab was not correlated with PD-L1 expression in Checkmate 025 while patients with positive PD-L1 expression seems to show more clinical benefit from immune-checkpoint inhibitors in Immotion150 and Checkmate 214 (Table 2).

**Table 2:** Summary of assays and response rates in immune checkpoint inhibitor trials.

**Drug**		**Antiboy for PD-L1 IHC assay**	**Definition of PD-L1 positivity**
Nivolumab (SA)	PD-1	Rabbit 28-8 (Dako)	PD-L1 ≥5% (TC)
Atezolizumab (SA)	PD-L1	Rabbit SP142 (Ventana)	IHC 1/2/3 (IC)
Nivolumab/Ipilimumab(C)	PD-1/CTLA-4	Rabbit 28-8 (Dako)	PD-L1 ≥1% (TC)
Atezolizumab/Bevacizumab (C)	PD-L1/antiVEGF	Rabbit SP142 (Ventana)	IHC 1/2/3 (IC)

Locally advanced or in metastatic renal cell carcinoma.

*TC, tumor cells; IC, immuno cells in the microenvironment; SA, single agent; C, combination of agents; IHC 1/2/3: IHC1 is ≥1%, IHC2 is ≥5%, IHC3 is ≥10%*.

## Predicitve immulogical biomarkers in renal cell carcinoma

Due to the increasing role of immunotherapy in clinical practice, the research of predictive response factors remains a critical but still unmet issue. A PD-L1 assessment on tumor cells through Dako PD-L1 IHC 28-8 pharmDx test was performed in both the Checkmate 025 and 214 trials. In Checkmate 025 nivolumab efficacy was not influenced by PD-L1 expression. However, patients expressing PD-L1 more than 1% (*n* = 181) showed a worse OS in both treatment arms thus suggesting a prognostic role more than a predictive one. On the other hand, an exploratory analysis of the Checkmate214 showed a PFS benefit favoring combination only in patients expressing PD-L1 (1% or greater). Survival and ORR advantages were maintained in all PD-L1 categories. However, patients with higher PD-L1 expression showed greater benefit with the immune-combination. Taking together, these results seem to confirm that PD-L1 IHC expression does not act as predictor of response in patients with metastatic ccRCC receiving ICI immunotherapy (12, 15, 28, 46, 54). Furthermore, intratumoral eterogeneity of PD-L1 expression is another issue to take into consideration. As demonstrated by López et al. a multisite tumor sampling strategy identified a greater number of positive cases compared to current tumor sampling protocols and a different pattern of PD-L1 expression with positive and negative regions in the same tumor (55).

As seen in other neoplastic diseases in which immunotherapy has been successfully tested, tumor mutational burden and non-synonymous mutation expression have been related to higher neo-antigens tumor expression and to favorable immunotherapy response. A rationale supporting additional research of this variable in RCC derives from the evidence that immunotherapy is associated to higher clinical benefit in worst risk categories of RCC, a clinical category of RCC in which high mutational load is present (30–32). Indeed, considering the subgroup analysis of the Checkmate 025 study and the significantly better results of nivolumab-ipilimumab combination in intermediate/poor risk patients in the Checkmate 214, it seems likely that tumors with worst clinical features are those that better respond to immune-checkpoint inhibitors and this may be due to a higher mutational load resulting in higher neo-antigen content. Unfortunately, differently than expected, mutational load does not seem to correlate with MSKCC or IMDC prognostic criteria (31). Moreover, no difference has been observed between clear cell and sarcomatoid components of different tumor samples, suggesting that the level of mutational load is not a variable associated to worst clinical features of the disease, hypothesis that clearly needs further investigation (33).

Concerning the correlation between mutational burden and response to ICI immunotherapy in ccRCC, de Velasco et al. carried out a whole exomes and transcriptomes sequencing of 9 patients with metastatic RCC receiving nivolumab. They discovered that RCC had relatively few non-synonymous mutations and neo-antigens and, surprisingly, that among patients receiving nivolumab non-synonymous mutations were significantly higher in non-responder patients (*n* = 6) compared to responder patients (*n* = 3) (34). Of note, they found a very impressive expression of immune-mediated genes (PD-L1, PD-L2, CTLA4, PD-1, PRF1, GZMA, BTLA, CD8A) in a single patient with PD-L1 expression less of 5% but >1% who showed an impressive complete response to nivolumab. Although no final conclusion could be resumed from this study due to the small number of patients explored it is probably that tumors mutational burden and non-synonymous mutations play a different role in ccRCC as compared to other disease, however, further large prospective trials might be necessary to confirm this hypothesis.

It is worth mentioning the IMmotion 151 trial, a randomized Phase III study of Atezolizumab plus Bevacizumab vs. Sunitinib in untreated metastatic RCC (35). Primary endpoints included PFS in PD-L1 positive patients and OS in an intention to treat analysis. The IMmotion 151 trial met its primary PFS endpoint in the PD-L1 positive patients with atezolizumab + bevacizumab compared to sunitinib with fewer high grade adverse reactions. This data does support atezolizumab + bevacizumab as first line therapy in metastatic clear cell renal cell (35, 56).

## Gene expression as predicitve immunological biomarkers in renal cell carcinoma

Regarding gene expression, several data seem to correlate the expression of specific classes of genes (especially DNA repair genes) to immunotherapy outcomes (30). Reportedly, the most frequent event involved in ccRCC is the loss of chromosome 3p, which is associated with the development of VHL, PBRM1, BAP1 and SETD2 alterations in about 90% of ccRCC cases. Together with KDM5C, PTEN, MTOR and TP53, these represent the eight most frequently altered genes in ccRCC (22, 23, 36). However, second most frequently mutated sub-network included AID1A, SMARCA4, and PBAF SWI/SNF chromatin remodeling complex. When mutations occur in chromatin regulators PBRM1, BAP1, and SETD2, several related genes showed altered expression as compared to VHL mutation (22, 23, 36). In particular, chromatin modification pathways interact with several genes involved in hormonal activity (*ESR1*), *RAS oncogene*, transcriptional output (*HIF1A, JUN, FOS, and SP1*), TGF-beta and especially DNA repair (*BAP1*) and immune-mediated signaling (*NFKB1 and IL-6*) (22, 23, 36). To date, no data about the correlation between gene expressions (especially DNA repair gene alterations and immune-related genes) are available, but this appears to be an attractive hypothesis to test mainly focused on the detection of predictive markers and the better understanding of mechanisms related to immune response in ccRCC (22, 23, 36).

The fundamental role of the gene alterations of PBMR1, BAP1 and SETD2 has been recently enforced by the findings presented at ASCO Annual Meeting 2018. The Spanish Oncology Genitourinary Group (SOGUG) presented the results of an observational prospective study collecting samples from 77 RCC patients treated with mTOR inhibitors everolimus or temsirolimus (79 and 21% of cases, respectively) (37). The study analysis included both IHC for p-S6, p-S6K1, p-AKT, p21, BAP1, and PBRM1 and NGS (next generation sequencing) for mutational analysis on key genes of mTOR pathway in RCC. Among enrolled patients, 87% had ccRCC histology; 60% had intermediate, 39% good prognosis, and 1% poor prognosis (MSKCC). No association between p-S6, p-S6K1, p-AKT, and p21 staining and response to temsirolimus/everolimus was reported. However, negative IHC expression for BAP1 and PBRM1 was associated with better mTOR inhibitor response (OR = 4.0, 95%CI = 1.4–11.9, *p* = 0.011 and OR = 3.9, 95%CI = 1.2–12.8, *p* = 0.025).

On the other hand, Bossé et al. reported on the prognostic value of genetic alterations resulting in loss of function (defined by the presence of pathogenic gene variant or 2 copy deletion) of VHL, PBRM1, BAP1, SETD2, TP53, and KDM5C, which are frequently mutated in metastatic RCC, in patients with ccRCC stratified by IMDC risk classification and treated with 1st line VEGFR tyrosine kinase inhibitors (38). Tumor samples were analyzed by NGS or whole exome sequencing (TCGA). Three hundred and eight patients were included; 21% of them with IMDC good risk features, 54% intermediate and 17% poor risk (8% unknown). The presence of gene alterations in VHL, PBRM1, SETD2, BAP1, TP53, and KDM5C was, respectively, 77, 43, 29, 19, 11, and 11%. Gene alterations in BAP1 were associated with worst OS (HR 1.7; 95%CI 1.1–2.5, *p* = 0.01), while alterations in PBRM1 and KDM5C were correlated with longer OS. Patients with tumors PBRM1 wild type and harboring gene alterations in BAP1 had worse OS (37 vs. 50 months, HR 1.9, 95% CI 1.2–2.8, *p* = 0.004). Interestingly, when IMDC stratified criteria were applied the genomic profile was prognostic only in patients with intermediate risk.

The advances in understanding the molecular landscape of RCC parallel with the progresses in the histopathological characterization of this neoplasm. The 2016 WHO classification of the tumors of the kidney (13) has identified new renal entities including hereditary leiomyomatosis and renal cell carcinoma syndrome–associated RCC, succinate dehydrogenase–deficient RCC, tubulocystic RCC, acquired cystic disease–associated RCC, and clear cell papillary RCC. The list of histologic categories includes also emerging entities, such as RCC associated with ALK gene rearrangements and thyroid-like follicular RCC (13). A more accurate identification of the different histological tumor categories represents another fundamental step forward for the selection of molecularly targetable approaches for patients with RCC thus enabling the possibility to selectively target the gene drivers of specific tumor variants (13). More recently, Chen et al. (16) surveyed 894 RCC cases for expression of genes involved in immune checkpoint pathways, including PD1 and PDL1 genes. Clear cell RCC subtypes had relatively high expression of several genes representing targets for immunotherapy, including PDCD1 (PD1), CD247 (CD3), PDCD1LG2 (PDL2), CTLA4 (CD152), TNFRSF9 (CD137), and TNFRSF4 (CD134). In addition, analysis of gene expression signatures and of DNA methylation signatures suggested greater levels of immune cell infiltrates, including T cells, within clear cell RCC relative to other RCC subtypes (16, 17).

Within clear cell-enriched (CC-e) RCC genomic subtypes, differential expression of specific checkpoint-related genes was observed mostly involving differences between CC-e.3 and CC-e.2 groups (more aggressive and less aggressive ccRCC categories, respectively). Compared to CC-e.2, CC-e.3 showed increased promoter methylation of miR-21 (MIR21) with corresponding decreased levels of the miR-21 target PTEN. In cancer, PTEN has an established role in intrinsic cellular control of PD-L1 (16, 17). Some other genes—including PDCD1, CTLA4, and TLR9—were associated with worse patient survival within ccRCC-associated cases; PDL1 expression was correlated with better patient survival, though this association was confounded by copy loss of 9p region associated with aggressive clear cell RCC and worse prognosis (18). In summary, better understanding the predictive and prognostic significance of PD1/PD-L1 expression and the identification of molecularly defined subtypes correlated with survival and response to therapy, represent quick steps toward implementing precision medicine in RCC via reducing the distance to the goal of identifying the best approach for a single RCC patient (28, 29, 40) (Table 3).

**Table 3:** Prognostic and predictive biomarkers in Renal Cell Carcinoma.

**Biomarker**	**Results**	**Association with**	**References**
IHC expression of p-S6, p-S6K1, p-AKT, and p21	NA	No association with response to temsirolimus/everolimus	(37)
Negative IHC expression for BAP1	OR = 4.0, 95% CI = 1.4–11.9, *p* = 0.011	Better mTOR inhibitor response	(37)
Negative IHC expression for PBRM1	OR = 3.9, 95% CI = 1.2–12.8, *p* = 0.025	Better mTOR inhibitor response	(37)
Gene alterations in BAP1	HR 1.7; 95% CI 1.1–2.5, *p* = 0.01	Worse OS	(38)
Gene alterations in PBRM1	HR = 0.6; 95%CI 0.4–0.8, *p* = 0.001	Better OS	(38)
Gene alterations in KDM5C	HR = 0.4; 95%CI 0.2–0.8, *p* = 0.007	Better OS	(38)
SETD2, TP53, and VHL	NA (*p* > 0.4)	Not associated with prognosis	(38)
PBRM1 wild type + gene alterations BAP1	37 vs. 50 months, HR 1.9, 95% CI 1.2–2.8, *p* = 0.004	Worse OS	(38)
PDCD1, CTLA4, and TLR9	NA	Worse OS	(16)
9p deletion	HR 4.323; *p* = 0.021 HR 4.603; *p* = 0.007	High risk of recurrence and RCC-specific mortality	(18)

## PD-L1 immunohistochemistry in RCC

Imunocheckpoint inhibitors (ICI) have marked a new paradigm in the treatment of RCC. The anti-PD1 drug nivolumab has been the first ICI drug to obtain approval by the FDA and European Commission for the treatment of RCC, and showed a significant OS benefit in patients with RCC that progressed following antiangiogenic therapy compared with everolimus (mTOR inhibitor) (26). Several other ICI compounds are currently under investigation for the treatment of RCC, alone or in combination with TKIs or other drugs (57–59).

Predictive biomarker research to select RCC patients eligible for ICI has mainly focussed on the PD1-PD-L1 axis detected by means of IHC. Low-to-no expression of PD-L1 on IC (immune cells) and TC (tumor cells) correlated with a trend toward lower response (PFS and OS) to the anti-PD-L1 drug atezolizumab compared with moderate to high PD-L1 expression levels (60). Updated analysis further confirmed the association between high PD-L1 expression and improved OS with atezolizumab treatment (57). For the anti-PD1 drug nivolumab, early data suggested a positive correlation between PD-L1 expression on TC and ORR (61–64). Data from the Checkmate 025 trial showed that higher levels of PD-L1 expression are associated with poorer survival in RCC, but did not support PD-L1 as a marker predictive of treatment benefit in RCC; a benefit was observed, however, with nivolumab irrespective of PD-L1 expression (62). Furthermore, PD-L1 seems to be a dynamic biomarker since prior exposure to VEGF and mTOR inhibitors modulates its expression which can be largely variable after therapy (64, 65). Notably, a significant number of patients with PD-L1+ RCC do not respond to PD-1 pathway blockade, suggesting that additional intra-tumoral factors may influence treatment outcome (64, 65). Based on recent data, PD-L1 could be a prognostic biomarker for the adverse clinic-pathologic features of RCC but may not be discriminant enough to be a predictive biomarker (64, 66, 67). Furthermore, it was found that PD-L1 staining is almost exclusively observed in the high-grade component of a tumor and additionally a discordant expression of PD-L1 between primary tumors and their metastases was detected in ~20% of cases (68). Similar heterogeneity has been observed between primary and metastatic tumor based on molecular analysis (69).

Other possible biomarkers like PD-L2 and CTLA4 are reported in literature, thus far without straightforward predictive value (57). Increased amounts of CD3^+^/CD8^+^ tumor-infiltrating T-cells have been reported after nivolumab treatment, but further research is needed to determine the biomarker-potential of this observations (70).

Recent data from a gene expression study on a small cohort of PDL1+ RCC patients treated with nivolumab identified a metabolic gene profile in the non-responding subgroup and overexpression of immunologic factors in the responding subgroup (71). Increasing mutational burden and neo-antigen formation have been associated with increased responsiveness to ICI in several other malignancies and recent data showed increased frequency of genomic alterations in RCC post-VEGFR therapy (72). These findings might explain the observed benefit of nivolumab post-VEGFR therapy and seem to correlate with the observation of lower response rates to nivolumab monotherapy in front line studies (70). A recent multilevel molecular analysis on the integrated TCGA RCC database showed relatively high expression of several genes representing targets for immunotherapy in ccRCC-associated molecular subtypes compared to other RCC subtypes, with additional differences within the several clear cell-enriched RCC genomic subtypes (16). These data also suggested greater levels of IC infiltrates within ccRCC relative to other RCC types (16). TCGA data suggest the hypothesis that clear cell-enriched RCC genomic subtypes would be most responsive to targeted immune checkpoints, hypothesis that awaits validation in prospective cohort series (16).

Several technical and biochemical issues are involved to explain the observed ambiguity of PD-L1 expression as predictor of response to ICI therapy in RCC. Differences in anti-PD-L1 antibody-clones, staining assays, tissue characteristics and scoring systems are amongst the major technical obstacles to overcome. The knowledge that PD-L1 expression is not binary, but instead shows a continuum with significant intratumour heterogeneity and therapy-induced changes, might even represent a bigger challenge for being an ideal biomarker (73, 74). The recent report on the presence of compensatory inhibitory pathways (VISTA) in the setting of immunotherapy in metastatic prostate further underlines the complexity to predict the therapeutic response based on a single biomarker like PD-L1 (74). Recent concordance studies on non-small cell lung cancer have shown only minimal differences in staining patterns between most of the different validated and commercially available anti-PD-L1 antibody clones (73, 75–78). These findings are encouraging, although clinical cross-validation data between the different assays are not available at this moment. High concordances between the different assays and between the pathologists within a single assay were only found for PD-L1 scoring TC and not in immune cells IC (75, 76). Concerning RCC this could be a critical point since PD-L1 expression in IC is used as a companion biomarker for some FDA-approved anti-PD-L1 drugs.

## MMR-deficiency and mutational load in RCC

Renal cell carcinoma are not considered to belong to the HNPCC (hereditary non-polyposis colon cancer) spectrum, but in sporadic RCC loss of MMR proteins is frequently observed, especially of MLH1 and MSH2 (79–81). Variable MMR gene alterations have been reported as underlying mechanisms, but others did not detect microsatellite instability (MSI) caused by either promoter hypermethylation or alteration of the coding region of MMR studied genes (81–83). The reduced MMR protein expression by IHC has been linked to RCC subtypes and might contribute to the respective different biological behavior (84). As addressed earlier in this review, MMR-deficiency is more and more recognized as an important biologic event in genitourinary cancers. MMR deficiency can occur in patients with Lynch syndrome (HNPCC) and in patients with sporadic MMR-deficient tumors (84). MMR-deficient tumors exhibit a higher rate of mutations (high mutational burden), which can result in the formation of neo-antigens to enhance the antitumor immune response (85). Furthermore, MMR-deficient tumors express different immune checkpoint ligands indicating that their active immune microenvironment is counterbalanced by immune inhibitory signals that resist tumor destruction (86). Recently reported data showing a better clinical response to the anti-PD-1 drug pembrolizumab in MMR-deficient patients support the hypothesis that MMR-deficient tumors respond better to anti-PD-1 therapy than do MMR-proficient tumors (87).

In RCC cancers, data on the relation between MMR-status and response to immunotherapy are still emerging (88, 89). Based on the promising results in patients with MMR-deficient cancers, FDA has recently approved pembrolizumab for the treatment of adult and pediatric patients with un-resectable or metastatic MMR-deficient solid tumors, irrespective of the tumor origin. In this context, MMR-deficient/MSI-H solid genitourinary tumors could be important candidates for anti PD-1 treatment. The reality might however be much more complex; for instance, several clinical trials have shown that some MMR-deficient tumors do not respond to immunotherapy, while mutations in other genes have also been linked to high mutational burden and upregulation of immune checkpoints (85). From a methodological point of view, there is an ongoing discussion and evolution in literature concerning the methodology to get reliable data on mutational load and MSI in a context of cost-efficiency and optimal logistics. Whole exome sequencing, T-cell receptor sequencing and targeted NGS can be used to assess mutational load (90) and promising data on novel platforms to detect MSI (e.g., MSI-Sensor and MANTIS) have recently been published (91). The detection of MMR-deficient tumors and the selection of those patients that will really benefit from immunotherapy remains an ongoing and challenging task.

## Biomarkers of acquired resistance

Despite the durable responses observed with immune checkpoint inhibition, nearly all patients will progress. A number of mechanisms have been identified including neo-antigen loss, upregulation of alternative immune checkpoints, loss of antigen presentation, and defective interferon signaling (92–94). A recent whole exome sequencing study on paired tumor samples prior to treatment with ICI and at the time of progression (*n* = 4, 2 treated with nivolumab/ipilimumab, 2 treated with nivolumab) was reported by Anagnostou et al. (92). Although they found an increase in total number of candidate neo-antigens, a subset of them was actually eliminated at the time of acquired resistance. In the four patients, there were 18, 10, 7, and 6 neo-antigens lost, and all of them had higher predicted MHC binding affinity. There were no copy number alterations of CD274 which encodes for PD-L1, PDCD1 encoding for PD-1, CTLA-4, JAK1, or JAK2. There were no genetic alterations in HLA or β2-microglobulin. They also evaluated clonal T-cell reactivity in three of these patients using peripheral blood mononuclear cells loaded with predicted neo-antigens cultured with purified T-cells. All patients showed clonal T-cell expansion to lost peptides and either no affinity or lower affinity for the wild type of the predicted neo-antigen (92). Neo-antigen loss and growth of a subclone lacking the neo-antigen eliciting the immune response are both potential explanations of this resistance mechanism, although the power of available information is limited. This mechanism of resistance underscores the rationale for using neo-antigen profiling as a predictive biomarker of benefit and also underscores the dynamic nature of these biomarkers.

Defects in the interferon-γ signaling pathway have also been identified as a major mechanism of resistance. Interferon-γ signaling plays a crucial role in the anticancer immune response. It has been shown to upregulate PD-L1 expression on TC and IC, to increase MHC Class I expression and promote antigen presentation, and recruit effector cells (92–97). It results in the downstream stimulation of JAK/STAT signaling pathway and expression of a number of anti-cancer genes (98). Mutations in JAK1/2 render cells insensitive to interferon-γ signaling, which results in escape from PD-L1 pathway inhibition and impairs the antitumor immune response. This has been identified as a mechanism of both primary and secondary resistance (99–101). Interferon-γ signaling has been demonstrated to increase expression of immune inhibitory molecules, such as indolaimine-2,3-deoxygenase (IDO) that can limit the anti-tumor response (101). Inhibition of IDO production is the subject of an ongoing clinical trial in combination with PD-1 immune checkpoint inhibition. Defects in antigen presentation, such as mutations in the β-2 microglobulin gene, have also been identified as a mechanism of resistance (100). Beta-2 microglobulin is essential for MHC class I molecule surface expression and a defect can block CD8-Tcell recognition. HLA loss is another potential mechanism of immune evasion and determining copy number alterations have been difficult due to the polymorphic nature of the locus. McGrahan et al. developed a computational tool using NGS data to determine HLA loss of heterozygosity in 100 early stage NSCLC patients. Interestingly, 40% of patients displayed HLA loss of heterozygosity and phylogenetic analysis shows that this is likely a later evolutionary event (102). TIM-3, LAG-3, and TIGIT are known alternative immune checkpoints that play a role in T-cell exhaustion and are expressed on tumor infiltrating lymphocytes (93). Koyama and colleagues identified TIM-3 to be upregulated in a murine model of NSCLC at the time of resistance to anti-PD-1 therapy and demonstrated a survival advantage with treatment using a TIM-3 blocking antibody. The authors additionally identified two patients with biopsies performed at the time of progression to anti- PD-1 therapy with increased TIM-3 expression (103). Novel therapeutic approach directed at these alternative immune checkpoints are the subject of ongoing clinical trials and are of potential relevance in RCC.

## Future perspective

The complex interplay of signaling pathways and inflammatory mediators seems to be crucial for RCC development and response to therapy (43, 44, 53). Immune cells including neutrophils, lymphocytes and macrophages have been implicated in promoting metastatic spread, tumor angiogenesis, in primary and acquired drug resistance, as well as in the formation of pre-metastatic niches (43, 44, 53). On this scenario, the checkpoint molecules have gained wide interest since the introduction of anti-CTLA-4 and anti-PD-1/PD-L1 agents into daily oncology practice (45). Beyond PD-1 and CTLA-4, a variety of molecules are emerging as potentially future therapeutic immunotargets in RCC (46). This list includes the V-domain immunoglobulin containing suppressor of T-cell activation (VISTA), which has been recently shown to exert its inhibitory activity by acting as a ligand on antigen presenting cells and as a receptor on T cells (104–106), chemokine receptors (45), the soluble lymphocyte-activation gene-3 (LAG-3), 4-1BB, B and T lymphocyte attenuator, and OX40 (CD134) (47).

Nowadays, there is not a clear-cut knowledge of the underlying mechanisms of immuno-checkpoint inhibitors-induced tumor response. To address this issue, Wei et al. investigated the effects of anti-PD-1 and anti-CTLA-4 inhibitors in human melanoma and murine tumor models (48). They first revealed that these agents are able to target distinct tumor-infiltrating T cell subpopulations. In particular, PD-1 blockade promotes the expansion of specific exhausted-like CD8-T cell population, while CTLA-4 blockade induces both an ICOS^+^ Th1-like CD4 effector subset and exhausted-like CD8-T cells (48). This evidence favors the combined use of current and probably future checkpoint inhibitors in cancer patients. These combinations, seems to be characterized by a tolerable safety profile (107). Tumor responsiveness may vary according to the mutational load and the expression of immunotargets in the tumor environment, which is variable in the different phases of RCC development and progression (49, 108–110). Based on this evidence, assessing the expression of PD-1/PD-L1 or other emerging immunotargets only at the diagnosis of metastatic disease may not reflect tumor dynamicity.

To improve the feasibility and reduce the clinical impact of re-biopsy, assessing biomarkers on circulating tumor cells (CTCs) or exosomes (111) may represent a not invasive strategy that can be performed several times during cancer therapy in order to reflect the changes occurred in the tumor environment. An early identification of validated biomarkers would be crucial to definitively place immunotherapy into the era of precision medicine and to optimize the cost-effectiveness of ICI agents in cancer patients (50, 112). In addition, the recent paper by Routy et al. showed that primary resistance to ICI can be correlated with abnormal gut microbiome composition (51). In this study, the effectiveness of PD-1 blockade resulted enhanced by transplanting fecal microbiota from responder cancer patients into germ-free or antibiotic-treated mice (51), thus representing another step forward on the way to personalized and precision immunotherapy in cancer patients.

Another factor that results of great relevance to improve the efficacy of ICI in RCC patients is the comprehension of the immunological effects of TKIs and mTOR inhibitors (53, 113). Actually, these agents can indirectly exert their anti-tumor activity by targeting immune cells in the RCC microenvironment (53), and this should be considered in order to combine or sequence them with currently available and probably future immunotherapies. For instance, sunitinib has been shown to inhibit the colony forming units driven by GM-CSF and FLT3 ligand FLT3L (114) as well as dendritic cell antigen-presentation (115) (by decreasing the secretion of cytokines and the expression of MHC and CD1a molecules), to suppress the myeloid-derived suppressor cells (MDSCs are involved in RCC progression and drug resistance), to enhance tumor cell sensitivity to NK cell killing (116) and to reduce the total count of CD3 and CD4 T cells and regulatory T cells (117, 118). On the other hand, pazopanib showed lower inhibitory potency and affinity against FLT3 and c-kit compared to sunitinib (119). Interestingly, we previously showed that axitinib can increase the surface NKG2D ligand expression, thus promoting NK cell recognition and degranulation in A-498 RCC cells in a ROS-dependent manner (120). At present, few evidences are available on the immunomodulatory effects of cabozantinib and lenvatinib, recently introduced into RCC clinical practice.

## Expert opinion and conclusions

Optimizing the combination between immunotherapy and target agents as well as the possible favorable sequence of treatment between these two classes of drugs remain open questions at this moment but ongoing studies support this as of great future potential. On this way we have only limited data provided from Immotion150 which demonstrated that association between a PD-L1 inhibitor and bevacizumab is feasible, well-tolerated, and results in an effective clinical benefit from our patients. Of relevance, is to note that most studies explored immunotherapy in patients with ccRCC and the role of ICI still remains unknown in mccRCC. Though, there are several questions that need to be answered, current data support that immunotherapy represents a revolution for the management of RCC resulting in a dynamic and evolving scenario in which more novelties will be shortly made available. Because the potentially deadly adverse effects from immune checkpoint inhibitors, gathering predictive information in RCC seems to be prudent. However, recent scientific insights indicate that a single biomarker for patient selection may not be feasible, given that immune responses are dynamic and evolve over time (121). Biomarker development for ICI drugs will require integration of multiple biologic components like PD-L1 expression, TILs, mutational load, and probably many others now considered emergent biomarkers.

## Executive summary

- Immunotherapy is gradually becoming a key factor in the therapeutic algorithm for patients with renal cell cancers at different stages of disease.
- The increasing knowledge on the genomic landscape of renal cell carcinoma supports stratification of patients for targeted therapies.
- A single biomarker for patient selection may not be feasible, given that immune responses are complex, dynamic and evolve over time.
- Biomarker development for ICI drugs will require integration of multiple biologic components like PD-L1 expression, TILs and mutational load.

## Next steps

- New methodological approaches likely based on digital pathology may be relevant since they allow objectively recognizing and quantitation of the biomarker and therefore might produce objective and reproducible cut-offs useful in patient's therapeutic stratification.
- Radiologic derived biomarkers, such as artificial intelligence derived, radiopharmaceutic, and liquid biopsy derived biomarkers, are likely to enter the biomarker-field in the next coming years.
- Large-scale biomarker-driven prospective trials with consensus methodologies on biomarker assessment and scoring are needed to reach clinical validation of different biomarkers, needed for a reliable single-patient appointment to the appropriate immunotherapy.
- Multidisciplinary approaches are needed to fully develop the current and future value of ICI in clinical practice.
- Better understanding of solid tumor genomics shows that also for RCC, combining targeted therapy with ICI has the potential to improve cancer outcomes, and that reliable biomarkers will be crucial for a stringent patient selection in trials of targeted and checkpoint inhibitor drugs and to apply novel therapeutic strategies aimed at restoring effective antitumor immunity in patients with cancer.

## Author contributions

AL-B, RM, MSc, and LC: conception and design; AL-B and VH: drafting the manuscript; AB, AC, and VH: review of the literature; AL-B, LC, RM, MSc, MSa, FM, VH, and TG: critical revision of the manuscript.

### Conflict of interest statement

The authors declare that the research was conducted in the absence of any commercial or financial relationships that could be construed as a potential conflict of interest.
